# The Psychoactive Agent Crocin Can Regulate Hypothalamic-Pituitary-Adrenal Axis Activity

**DOI:** 10.3389/fnins.2017.00668

**Published:** 2017-12-01

**Authors:** Sara Asalgoo, Mahdi Tat, Hedayat Sahraei, Gila Pirzad Jahromi

**Affiliations:** ^1^Behavioral Sciences Research Center, Baqiyatallah University of Medical Sciences, Tehran, Iran; ^2^Applied Virology Research Center, Baqiyatallah University of Medical Sciences, Tehran, Iran; ^3^Neuroscience Research Centre, Baqiyatallah University of Medical Sciences, Tehran, Iran

**Keywords:** post-traumatic stress disorder, hypothalamic-pituitary-adrenal axis, Crocin, dopamine-dependent behaviors, Corticosterone

## Abstract

Post-traumatic stress disorder (PTSD) occurs following life-threatening events. The activity of the hypothalamic-pituitary-adrenal (HPA) axis, which serves as the first line of defense against stress, is dysfunctional in this disorder. The current study aimed to investigate the role of Crocin in normalizing HPA function in an animal model of PTSD induced by electric foot shock. Rats were treated with Crocin 5 min prior to stress induction. The stimulus was re-introduced after 21 days, and we measured individual behaviors such as sniffing, rearing, grooming, and freezing. Enzyme-linked immunosorbent assays were performed to measure plasma levels of Corticosterone. On day 28, after rats were weighed and sacrificed, the adrenal and thymus glands were removed and subjected to real-time polymerase chain reaction to quantify the gene expression of corticotrophin-releasing hormone (*CRH*), glucocorticoid receptor (*GluR*), and arginine vasopressin (*AVP*). Our results demonstrate that rats re-exposed to a stressor developed characteristic symptoms of PTSD, but these were attenuated by Crocin. Treated rats showed significant changes in *CRH* expression in the hypothalamus, *GluR* expression in the pituitary, plasma Corticosterone levels, and freezing behavior. Together, these findings suggest that Crocin can regulate HPA axis activity in PTSD. It may serve an appropriate treatment for subjects who experience a traumatic event.

## Introduction

Epidemiologic studies have reported that about 4–23% of people with traumatic damages manifest post-traumatic stress disorder (PTSD) symptoms including re-experiencing, avoidance, and hyperarousal for at least 1 month (American Psychiatric Association, 2013; Arnberg et al., 2013; Zhou et al., 2013). Biological alterations may underline PTSD symptom onset and persistence (Clément et al., 2002; Ozer et al., 2003; Segman et al., 2005). The hypothalamic-pituitary-adrenal (HPA) axis is the main neuroendocrine arm of the stress response. It includes corticotrophin-releasing hormone (CRH) release from the paraventricular nucleus (PVN) of the hypothalamus into the hypophyseal-portal circulation, stimulating the anterior pituitary gland to release adrenocorticotropic hormone (ACTH) and ultimately glucocorticoids from the adrenal cortex. This mechanism maintains homeostasis in stressful environmental conditions (Jacobson, 2005). Neuroendocrinologic studies have reported three common features in patients with PTSD: (1) decreased plasma cortisol; (2) higher CRH in the cerebrospinal fluid (CSF) and plasma; and (3) increased inhibition of the hypophysis-adrenal system (HAS), which leads to reduced cortisol levels by enhancing negative feedback (Carvalho et al., 2008). CRH and vasopressin are the most important mediators of the HPA axis' effects on neuroendocrine signaling.

Studies have shown that AVP expression in paraventricular neurons and V1b receptor density in pituitary corticotroph significantly increased in response to chronic stress (Kovács and Sawchenko, 1996; Aguilera and Rabadan-Diehl, 2000). These findings indicate that AVP plays an important role in responding to stress by maintaining the ACTH sensitivity to stressors during chronic stress (Smith and Vale, 2006).

Considering the synergistic effect of corticotrophin and vasopressin in response to stress, as well as the role of stress in PTSD development, these neurohormones likely play a role in PTSD pathogenesis. ACTH is a key mediator in glucocorticoid secretion from the adrenal cortex. Glucocorticoid hormone release, (cortisol in humans and Corticosterone in rats), is the final effect of HPA axis activity, and they play a significant role in the stress response (Yehuda, 2009). Studies of patients with PTSD showed increases in the number and sensitivity of glucocorticoid receptors (Yehuda et al., 1995; Rohleder et al., 2004), as well as greater inhibition of cortisol and adrenocorticotrophin following dexamethasone treatment (McFarlane et al., 2011).

Crocin (Crocin di-gentiobiose ester) is an isolated chemical compound that belongs to a group of commercial carotenoid derived from the stigma branches of dried saffron. Crocin can improve learning and memory (Ebadi, 2006) and may prevent neurodegenerative disorders including Alzheimer's disease (Rohleder et al., 2004). It is not mutagenic (Papandreou et al., 2006) and prevents alcohol-induced disorders of memory and learning (Papandreou et al., 2006). Its mechanism is thought to be prevention of the inhibitory effect of ethanol on N-methyl-D-aspartate (NMDA) glutamate receptors in the hippocampus (Ebadi, 2006).

However, it is not clear that what cellular and molecular mechanisms are underlying the Crocin action on PTSD. In addition, it is not clear that what would happen if the stress paradigm became shorter in time duration. We aimed to evaluate the role of Crocin on HPA axis activity. To this end, we evaluated the effect of intracerebroventricular (ICV) infusion of Crocin on expression of genes involved in HPA pathway, plasma Corticosterone levels, and behavioral symptoms were evaluated in an electric foot shock rat model of PTSD (Langevin et al., 2010).

## Materials and methods

### Animals and treatments

**Figure 1:**
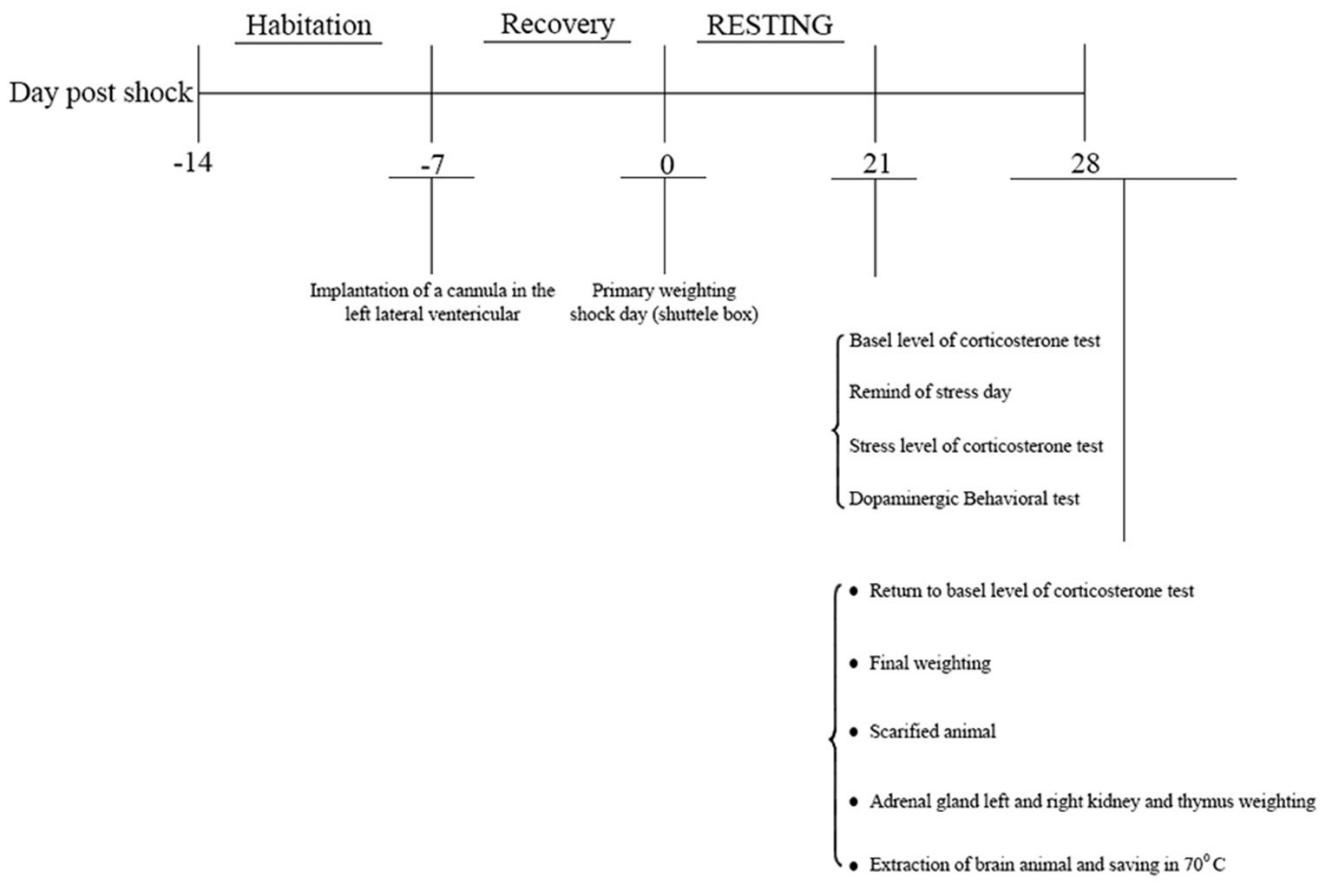
Study protocol.

This study was approved by the joint council of the centers for the study of behavioral sciences and neuroscience on 1/21/2014. Indeed, it was reviewed by research committee of Baqiyatallah University which approved all protocols as well. Male Wistar rats (weight: 180–250 g, 8–10 weeks old, Pasteur Institute, Tehran, Iran) were procured 1 week prior to the experiments and housed 3–4 per cage at 22 ± 2°C temperature under a 12:12-h light/dark cycle with free access to water and food, except for during the experiment. Animals were housed in the laboratory for 28 days, and experiments were conducted during daylight hours under standard conditions. Animals were randomly divided into six groups were as follows: 1-Negative control (no manipulation), 2- Sham (surgery without cannula implantation), 3- Drug control, 4- positive control (PTSD without surgery), 5- PTSD (surgery) + normal saline, 6-PTSD (surgery) + Crocin Rats were evaluated 7 days after surgery (Figure 1).

### Stereotaxic surgery

**Figure 2:**
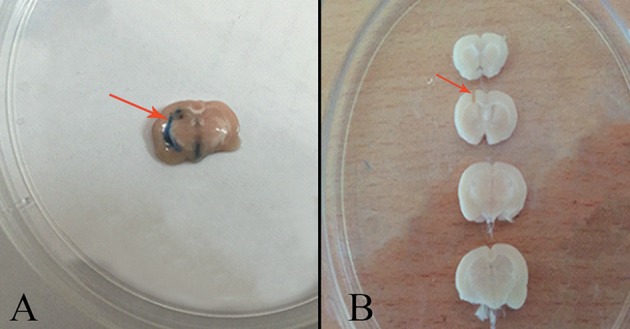
**(A)** Area of trypan blue injection into the left lateral cerebroventricle (arrow). **(B)** The area of cannula implantation into the left lateral cerebroventricle (arrow).

After induction of anesthesia with 70 mg/kg ketamine and 10 mg/kg xylazine, animal heads were placed in stereotaxic apparatus equipped with a 23-gauge stainless steel cannula (Stolting Instruments, Wood Dale, IL, USA) (Sahraei et al., 2012). The area of infusion was determined and marked following the coordinates given in the Paxinos and Watson Atlas (1987) for the left lateral cerebroventricle beyond a guide cannula: −9 mm posterior to bregma, ±1.5 mm from the sagittal line to lateral, and 3.5 mm down from the dura, and −3.3 mm from the maxillary tooth row. The guide cannula was inserted into the skull and fixed onto the head with dental cement. Animals were allowed to recovery for 1 week after surgery. For the stress protocol, 5 min prior to the electric shock, the infusion (10 μg/mL) (Hooshmandi et al., 2011) was gently administered over 1 min beyond a guide cannula with a 10-μL Hamilton syringe attached to the polyethylene tube. Then, the Hamilton syringe and polyethylene tube were removed. A 10 μg/mL Trypan blue solution was injected intracerebroventricularly to confirm the cannula area of implantation into the left lateral cerebroventricle (Figure 2).

### Establishing an electric foot shock rat model of PTSD

A shock box was employed to induce electric foot shock and develop an acute PTSD model. A transparent Plexiglas shock box (Borj Sanat Co., Tehran, Iran) with 9 chambers (16 × 16 cm) was used. The chamber floor was covered with 4-mm diameter stainless steel rods every 2 cm. The bottom of the box had 2.5-cm diameter holes for olfactory and auditory stimuli delivery. The rods embedded into the floor were connected to an electricity generator that delivered current with adjustable frequency, strength, and time. The PTSD group received 2-mA electric shocks for 1 s, every 30 s for 5 min. Afterwards, rats remained in the box for 3 min before they were transferred to the laboratory (Mikics et al., 2008).

### Stress re-exposure

Animals were transferred to the laboratory 21 days after the first acute stress for stress re-exposure (Mikics et al., 2008; Zoladz et al., 2012), and were separately placed in the shock box for 5 min without delivering electric shock. Overt behaviors (freezing, sniffing, rearing, and grooming) were recorded with a digital camera. The behavior of each group was separately evaluated and analyzed (Fedele et al., 1998). The animals were returned to the laboratory after re-exposure.

### Plasma corticosterone measurement

The plasma Corticosterone level of all groups (*n* = 4 per group) was measured. Accordingly, 2-mL blood samples were collected from the corner of the rats' eyes before (basal) and after re-exposure (stress) and on day 28 of the experiment (return toward basal level). The blood samples were centrifuged at 3,000 rpm for 8 min, and serum samples were separated and stored at −70°C until use. Corticosterone was assessed with a DRG® enzyme-linked immunosorbent assay (Cat. # EIA 4164; DRG, Springfield Township, NJ).

### Weight measurements

Animals' weights (*n* = 7–8 per group) were assessed on days 1 and 28 in the control, PTSD and Crocin groups. After induction of anesthesia, the adrenal and thymus glands were removed and weighed according to previous studies (Ulrich-Lai et al., 2006; Gruver and Sempowski, 2008).

### Quantitative real-time reverse transcription polymerase chain reaction (RT-PCR)

Total RNA was extracted from pituitary and hypothalamus samples by using total RNA purification kit (Gene All® Hybrid-R™). The concentration of extracted total RNA was measured by a NanoDrop spectrophotometer (Thermo Fisher, Waltham, MA, USA) at 260/280 ratio (2.2). Primers were designed by CLC Sequence Viewer 6, Oligo 7, and GENRUNR software for the following genes: β-actin (NM_007393), *CRH* (NM_205769.3), glucocorticoid receptor (*GluR*, NM_DQ504162), and arginine vasopressin (*AVP*, NM_009732) (Table 1). To design AVP primers, introns were excluded and exons were put together; therefore, 2 different bands of RNA and DNA were generated from a primer. A Hyper Script™ reverse transcriptase kit (GeneAll®, Seoul, South Korea) was used to synthesize cDNA. On day 28 post-shock, the quantitative expression of *CRH* and *AVP* genes in the hypothalamus and *GluR* in the pituitary was evaluated with an ABI7500 Real Time System (Applied Biosystems, Foster City, CA, USA). The Real time RT-PCR amplification was performed using Composition of Real Q plus 2x Master Mix Green, Low Rox™ (Cat #A324402; AMPLIQON, Odense, Denmark). The cycling conditions were as follows: 15 min at 95°C, followed by 40 cycles of 20 s at 95°C, and 60 s at 60°C. The fluorescence level was analyzed at the end of each 60°C step. The amplification of each gene was evaluated through melting curve and visualization on gel electrophoresis. Quantitative assessment of genes expression was performed using the 2^−ΔΔCT^ method (Winer et al., 1999).

**Table 1:** Forward and Reverse Sequences of RT-PCR Primers.

**Target gene**	**Sequence length (bp)**	**Primer**	**References**
*β-actin*	149	F: GGGAAATCGTGCGTGACATC	Current study
		R: GAACCGCTCGTTGCCAATAG	
*CRH*	150	F: CTCTCTGGATCTCACCTTCCAC	Chen et al. (2012)
		R: CTAAATGCAGAATCGTTTTGGC	
*GluR*	98	F: AGTGATGGGGAATGACTTGG	Current study
		R: GGAAGAAAGCATTGCAAACC	
*AVP*	185	F: AGGGCAGGTAGTTCTCCTCC	Current study
		R: CTTCCAGAACTGCCCAAGAGG	

### Statistical analysis

The quantitative findings are presented as mean±SEM. One-way analysis of variance and Tukey's tests were used to compare intergroup means. *P* < 0.05 was considered significant.

## Results

### Overt behavioral identification

Overt behaviors were previously observed by a number (Ozer et al., 2003; Segman et al., 2005; American Psychiatric Association, 2013) of colleagues unfamiliar with the behaviors. So the size effect was resolved.

### Behavioral results in non-treatment PTSD model animals

Forty-eight rats were tested in the model, and 32 were exposed to electric foot shock.

The time required for freezing was significantly increased [*F*_(5, 38)_ = 6.03 *p* < 0.001, Partial Eta Squared = 0.44], whereas the time required for behaviors such as sniffing [*F*_(5, 38)_ = 6.14, *p* < 0.001, Partial Eta Squared = 0.44], rearing [*F*_(5, 38)_ = 5.31, *p* < 0.001, Partial Eta Squared = 0.41], and grooming [*F*_(5, 38)_ = 16.57, *p* < 0.001, Partial Eta Squared = 0.68] was remarkably decreased in PTSD animals (Table 2).

**Table 2:** Effects of ICV administration of crocin on dopamine-dependent behaviors in rats 21 days after electric foot shock stress termination.

**Behavior/Group**	**Freezing**	**Rearing**	**Sniffing**	**Grooming**
Negative control	10 ± 3.46	8 ± 1.29	14.66 ± 2.12	25.16 ± 2.28
Sham	27.66 ± 6.79	3.5 ± 1.05^**^	8.16 ± 0.9^**^	12.83 ± 2.21^***^
Drug-Control	16.16 ± 5.46	4 ± 0.57^*^	6.83 ± 0.47^**^	16.50 ± 0.88^*^
Positive control	153.71 ± 49.12^*^	3.14 ± 0.34^**^	6.71 ± 0.83^***^	9.14 ± 0.85^***^
PTSD+saline	149.85 ± 41.02^*^	3.57 ± 0.64^**^	6.28 ± 1.08^***^	9.28 ± 1.42^***^
PTSD+Crocin	24.14 ± 4.45^+^^#^	4.57 ± 0.61^*^	6.71 ± 1.14^***^	14.71 ± 1.83^**^

*Data are shown as mean ± SEM, n = 8 per groups*.

* *P < 0.05*,

** *P < 0.01*,

*** *P < 0.001 showed significant different in sham, positive control, positive+ saline, and PTSD+Crocin in compared to negative control group in rearing, sniffing and grooming behaviors but there was not any significant different in freezing behavior between treatment group and negative control group*.

+ P < 0.05 and

# *P < 0.05 showed a significant decrease in freezing behavior in PTSD+Crocin group compared to positive control and PTSD+saline respectively*.

**Figure 3:**
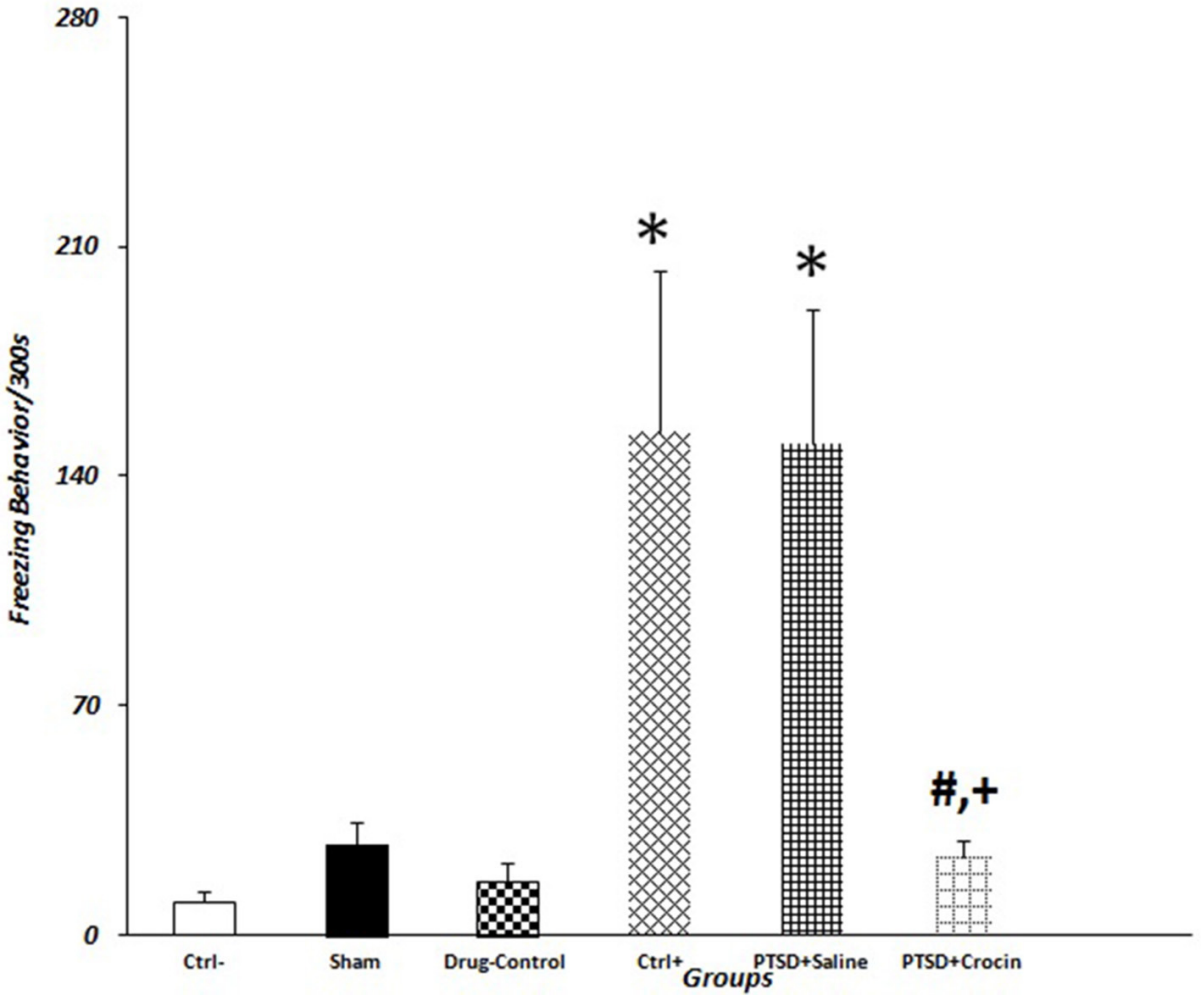
Data are shown as mean ± SEM, *n* = 8 per groups. Exploratory behavior was measured on day 21 post-shock at the re-exposure time, and freezing behaviors were quantified. *P < 0.05 showed a significant increase in freezing time in positive control and the PTSD+saline groups compared to the negative control group. ^+^*P* < 0.05 and ^#^*P* < 0.05 showed a significant decrease in freezing time in the PTSD+ Crocin group compared to the positive control and the PTSD+saline groups, respectively.

The mean value of freezing time in PTSD model animals including positive control (153.71 ± 49.12) and PTSD+saline (149.85 ± 41.02) groups were significantly greater than in the negative control group (10 ± 3.46) (*P* < 0.05). There were significant decreases in the mean number of rearing behaviors in rats with PTSD in the positive control (3.14 ± 0.34) and PTSD+saline (3.57 ± 0.64) groups compared with the negative control group (8 ± 1.92) (*P* < 0.01). Regarding grooming behavior, the mean numbers of times that rats touched their head and face in the group with PTSD in the positive control (9.14 ± 0.85) and PTSD+saline (9.28 ± 1.42) groups were significantly fewer than observed in the negative control group (25.16 ± 2.48) (*P* < 0.001). There were also significant decreases in the mean number of times that rats sniffed around purposefully in the PTSD with positive control (6.71 ± 0.83) and PTSD+saline (6.28 ± 1.08) groups compared with the negative control group (14.66 ± 2.14) (*P* < 0.001) (Figure 3).

As it is shown in Table 2, it is obvious that animals in the sham group exhibited overt behavior (sniffing, rearing and grooming) that was statistically different from negative controls. However, these animals did not exhibit any statistically significant differences in freezing behavior (*P* = 0.9) as compared to the negative control group. One explanation for this difference could be related to the fact that surgical operation is considered as a kind of stress, which can include alterations in overt behavior (Cesarovic et al., 2014).

### Behavioral results in pretreatment PTSD model rats

Eight animals were tested in this model with ICV infusion of Crocin given 5 min before electric shock exposure. Treatment reduced the mean value of freezing time (24.14 ± 4.45) to a level similar to the negative control group. However, sniffing, grooming, and rearing behaviors were not similar compared with the negative control group (all *P* > 0.05, Table 2).

### Corticosterone results

#### Basal levels

**Figure 4:**
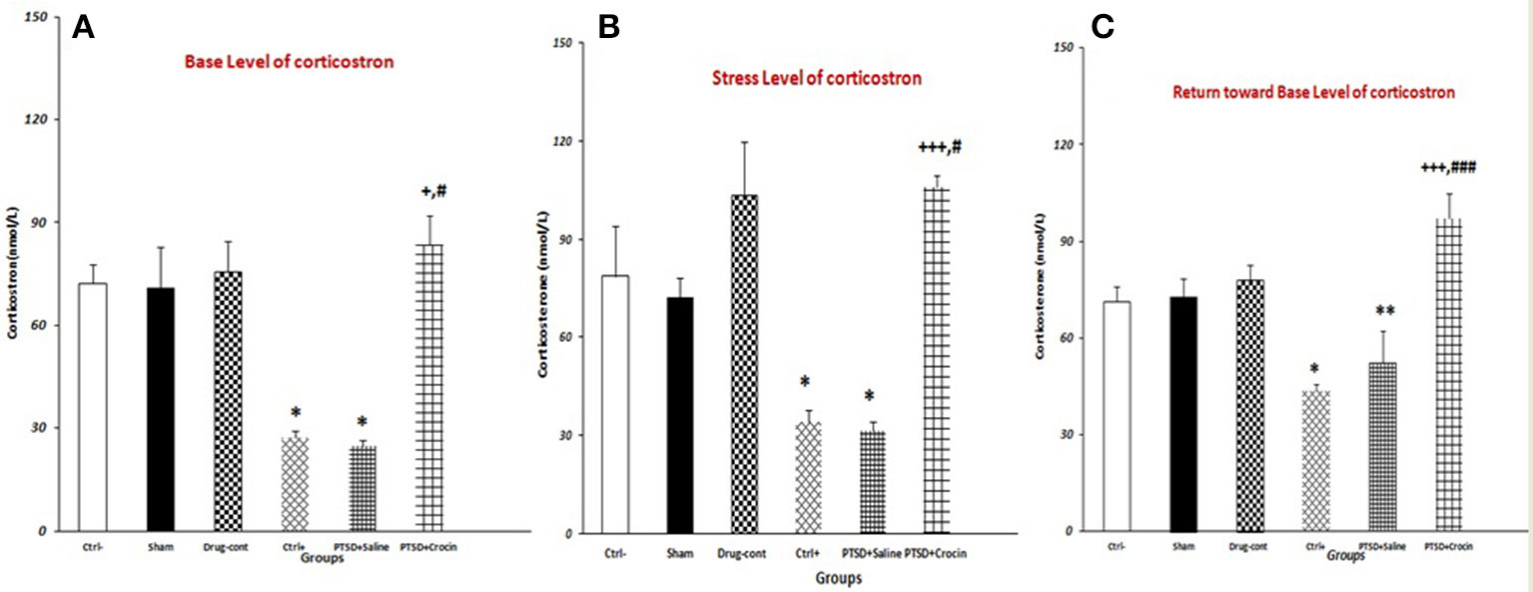
Data are shown as mean ± SEM, *n* = 4 per groups. Evaluation of serum corticosterone under three conditions (basal, stress, return toward basal). **(A)** Evaluating basal serum corticosterone before re-exposure time; a significant increase was observed in the PTSD+Crocin group compared with the positive control and PTSD+saline groups (^+^*P* < 0.05, ^#^*P* < 0.05) and significant decreases in the positive control and PTSD+saline groups compared with the negative control group (*P < 0.05). **(B)** Stress-induced corticosterone increased post-shock; there was a significant increase in the PTSD+Crocin group compared with the positive control (^+++^*P* < 0.001) and PTSD+saline groups (^#^*P* < 0.05) and significant decreases in the positive control and PTSD+saline groups compared with the negative control group (*P < 0.05). **(C)** At a later time point, there was a significant increase in PTSD+Crocin group compared with the positive control (^+++^*P* < 0.001) and PTSD+saline (^###^*P* < 0.001) groups and significant decreases in the positive control (*P < 0.05) and PTSD+saline (**P < 0.01) groups compared with the negative control group.

PTSD significantly decreases the basal serum Corticosterone level, which was abolished, when ICV infusion of Crocin took place [*F*_(5, 18)_ = 15.28, *p* < 0.001, Partial Eta Squared = 0.80] (Figure 4). There were significantly lower serum Corticosterone levels in rats with PTSD in the positive control (27.20 ± 1.93) and PTSD+saline (24.74 ± 1.70) groups compared with the negative control group (72.33 ± 5.6) (both *P* < 0.05). Notably, serum Corticosterone was significantly higher in the PTSD+Crocin group (83.50 ± 8.52), compared with the positive control and PTSD+saline groups (both *P* < 0.05) (Figure 4).

#### Stress levels

Moreover, PTSD caused a significant decrease in the serum Corticosterone after stress re-exposure, and ICV administration of crocin increases the hormone [*F*_(5, 18)_ = 14.61, *p* < 0.001, Partial Eta Squared = 0.80]. We measured significantly lower serum Corticosterone in the positive control (34.40 ± 3.26) and PTSD+saline (31.25 ± 2.39) groups compared with the negative control group (78.66 ± 15.45) (both *P* < 0.05). Stress-induced Corticosterone was also significantly higher in the PTSD+Crocin group (105.75 ± 3.79) compared with the positive control (*P* < 0.001) and PTSD+saline groups (*P* < 0.05).

#### Assessment on day 28

PTSD induced a significant decrease at the serum Corticosterone in return toward basal level [*F*_(5, 18)_ = 21.33, *p* < 0.001, Partial Eta Squared = 0.85]. In rats with PTSD and without Crocin, significantly lower serum Corticosterone was noted in the positive control (43.60 ± 2.20) (*P* < 0.05) and PTSD+saline (35.75 ± 4.49) (*P* < 0.01) groups compared with the negative control group (71.33 ± 4.66). A significant increase and then return toward basal level was observed in the PTSD+Crocin group (97.25 ± 7.67) compared with the positive control and PTSD+saline groups (both *P* < 0.001).

Our results showed that following Crocin treatment in rats with PTSD, serum Corticosterone measurements at three time points (basal level, stress level, return toward basal level) were similar to that of the negative control group. A significant decrease in the post-shock level of serum Corticosterone was observed in rats with PTSD and without Crocin (Figure 4).

### Weighing results

No significant differences were detected in different between first and last weight body [*F*_(5, 40)_ = 0.30, *p* = 0.9] or the right adrenal [*F*_(5, 40)_ = 1.38, *p* = 0.25], left adrenal [*F*_(5, 40)_ = 1.41, *p* = 0.23] and thymus [*F*_(5, 40)_ = 1.69, *p* = 0.15] weights of PTSD model animals without and with treatment compared with the negative control group (Table 3).

**Table 3:** Body and tissue weight changes.

**Group**	**Whole body (g) Mean ± SEM**	**Right adrenal (mg), Mean ± SEM**	**Left adrenal (mg), Mean ± SEM**	**Thymus (mg), Mean ± SEM**
Negative control	37 ± 7.1	0.009 ± 0.000	0.012 ± 0.000	0.29 ± 0.03
Sham	26 ± 6.8	0.013 ± 0.001	0.017 ± 0.002	0.29 ± 0.03
Drug control	35 ± 5.2	0.015 ± 0.001	0.010 ± 0.002	0.26 ± 0.18
Positive control	33 ± 4.6	0.016 ± 0.001	0.016 ± 0.002	0.28 ± 0.02
PTSD+saline	33 ± 8.2	0.015 ± 0.002	0.015 ± 0.002	0.31 ± 0.02
PTSD+Crocin	34 ± 2.4	0.014 ± 0.002	0.018 ± 0.003	0.35 ± 0.01

### *CRH* expression

**Figure 5:**
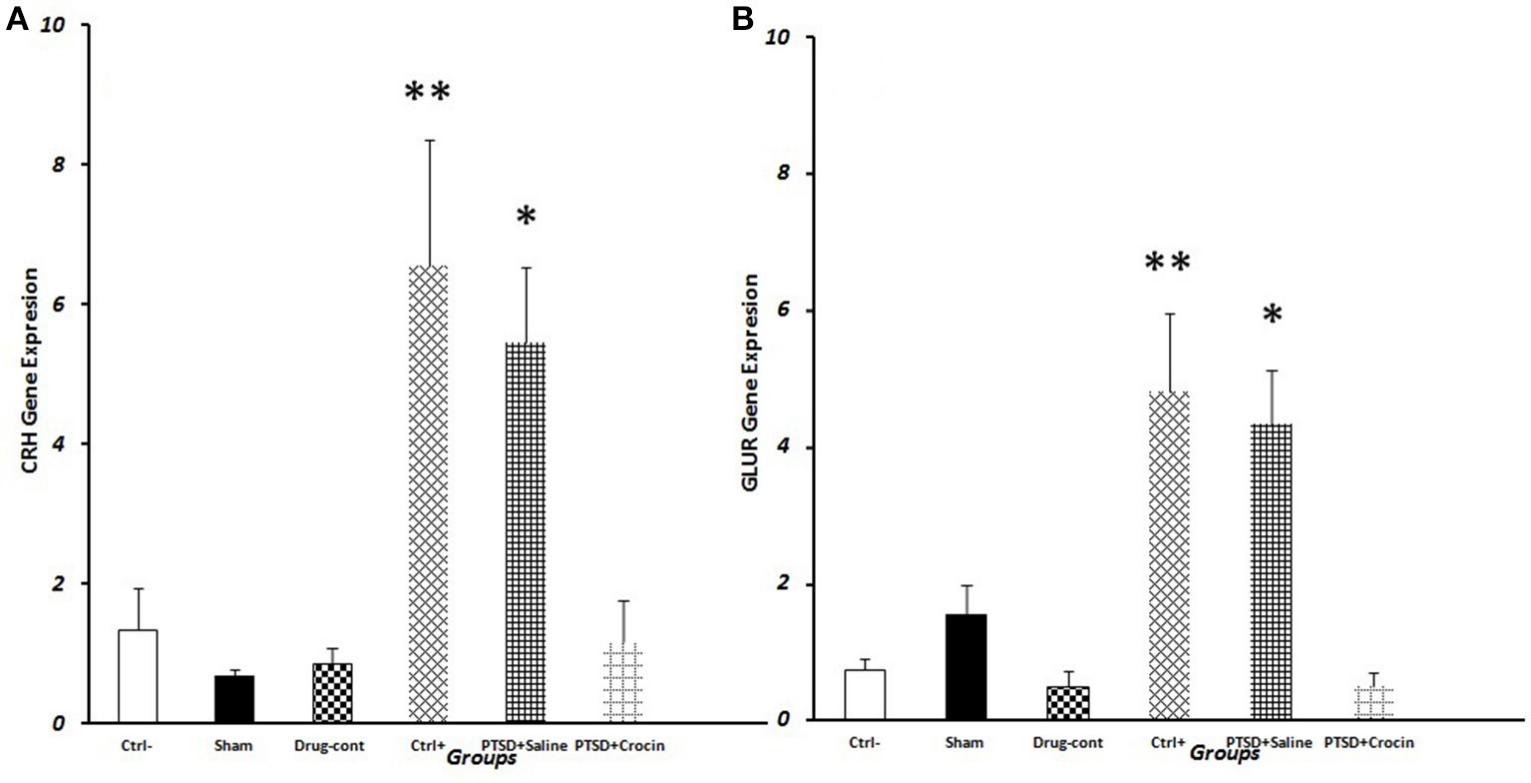
Data are shown as mean ± SEM, *n* = 4 per groups. RT-PCR measurement of gene expression on the day 28 post-shock. **(A)** Hypothalamic CRH expression post-shock; *P < 0.05, **P < 0.01 compared to the negative control group. **(B)** Pituitary GluR expression post-shock; *P < 0.05, **P < 0.01 compared to the negative control group.

We observed significantly greater *CRH* gene expression in PTSD model animals [*F*_(5, 18)_ = 7.763, *p* < 0.001, Partial Eta Squared = 0.68] including the positive control (6.56 ± 1.80) (*P* < 0.01) and PTSD+saline groups (5.45 ± 1.09) (*P* < 0.05) compared with the negative control group (1.34 ± 0.59). Also, in the PTSD+ Crocin group (1.17 ± 0.6) (*P* < 0.05), quantitative expression of the CRH gene significantly decreased compared to positive control and PTSD +saline groups, while did not significant change compared to the negative control group (Figure 5).

### *AVP* expression

There were no significant differences in hypothalamic AVP expression among any of the treatment groups [*F*_(5, 18)_ = 0.54, *p* = 0.7].

### *GLuR* expression

We observed significantly greater mean *GluR* expression [*F*_(5, 18)_ = 10.49 *p* < 0.001, Partial Eta Squared = 0.74] in PTSD groups that in the positive control (4.8 ± 1.14) (*P* < 0.01) and PTSD+saline (4.33 ± 0.79) (*P* < 0.05) groups compared to the negative control group (0.74 ± 0.16). Conversely, *GluR* expression in PTSD model rats infused with Crocin (0.49 ± 0.2) was similar to that in the negative control group (Figure 5).

## Discussion

The aim of the present study was to test the efficacy of Crocin as a treatment for PTSD using an animal model. The system in our study re-exposed rats to stress conditions. On the day 21 post-shock, serum Corticosterone levels were reduced in rats with PTSD; gene expression of *CRH* and *GluR* increased in the hypothalamus and pituitary, respectively; and overt behavioral changes were observed. Our results indicated that left lateral ICV infusion of Crocin can reduce *CRH* and *GluR* expression in specific brain areas. It can also increase serum Corticosterone at different time points and reduce stress-induced behaviors such as freezing in rats with PTSD. Several studies conducted on acute and chronic stresses reported that stressing factors can affect nervous, endocrine, and behavioral systems and induce physiological adaptations to preserve homeostasis (Figueiredo et al., 2003).

CRH regulates stress responses through activation of the HPA axis, which results in glucocorticoid release. CRH is also secreted from hippocampus and amygdale, where it plays the role of a neurotransmitter agent and responds to behavioral and autonomic stresses (Aguilera, 1998; Chen et al., 2004). High levels of CRH in the anterior pituitary desensitize CRH receptors, decreasing their responses (Dautzenberg et al., 2001; de Kloet et al., 2006).

Increased CRH was also observed in our model of PTSD. Stress behaviors were observed, while there was a significant decrease in *CRH* expression in the treatment-Crocin group compared with those with no treatment. Therefore, it is the indication of Crocin effect through the HPA axis to reduce hypothalamic CRH. A study evaluating the effect of saffron extract and Crocin on PTSD in rats reported corroboratory results (Sahraei et al., 2012). A likely mechanism of Crocin in regulating CRH secretion might be through alteration of a glutamate-dependent mechanism. Researchers indicated that glutaminergic innervations of the PVN changes under conditions of chronic stress and may be involved in sensitizing HPA axis responses (Zelena et al., 1999; Pistovcakova et al., 2005; Popoli et al., 2012). On the other hand, inhibition of NMDA receptors (NMDARs) by some components in saffron extract has been shown (Lechtenberg et al., 2008). Moreover, it has been indicated that saffron extract can increase brain glutamate levels (Ettehadi et al., 2013). Other studies reported that glutamate receptors are in post-synaptic and presynaptic forms, with presynaptic inhibitory receptors playing an important role in regulating glutamate release (Yuen et al., 2012). Collectively, the data suggest that glutamate plays a complex role in exciting CRH neurons, acting at multiple levels to both drive HPA axis responses and limit over activation (Feldman and Weidenfeld, 1997; Ulrich-Lai et al., 2011). Considering the inhibition of *CRH* expression in rats treated with Crocin, it seems that the Crocin plays a genomic role, in addition to its inhibitory effects on NMDA receptors. The finding of reduced *CRH* is likely relevant to the mechanism of PTSD, but studies in primates or humans are needed to clarify this pathway.

Vasopressin is a neuropeptide expressed in different hypothalamic nuclei such as the PVN supraoptic nucleus (SO) and suprachiasmatic nucleus (SC). Chronic stress increases vasopressin synthesis, which is secreted in the pituitary portal system and stimulates the HPA axis, resulting in ACTH release (Dorsa et al., 1988; Hernando et al., 2001). A recent study showed decreased vasopressin in the magnocellular area of the PVN after a forced swimming stress (Ettehadi et al., 2013). Vasopressin can improve the inhibitory feedback of glucocorticoids on ACTH release and stimulate corticotrophin expression to respond to new stresses and reactivate the HPA axis (Hauger and Dautzenberg, 2000).

We did not find significant differences in AVP expression between the PTSD and non-PTSD groups, which may be due to the different mechanisms of parvocellular and magnocellular neurons in the stress response in rats with PTSD. It may also depend on the timing of stress exposure and re-exposure. However, further studies to evaluate the status of *AVP* gene expression under PTSD conditions are recommended to clarify this issue.

A major neuroendocrine finding is that PTSD patients exhibit abnormally high GluR sensitivity resulting in HPA over suppression due to corticosteroid negative feedback (Yehuda, 2009). We observed increased *GluR* expression in the pituitary of rats with PTSD compared with animals treated with Crocin. The results indicate that Crocin may recalibrate GluRs and change the mechanism of response to Corticosterone by decreasing *GluR* gene expression. These results hint at the mechanism of the brain's response to PTSD; however, human studies are recommended to precisely investigate the relationship between *GluR* gene expression and PTSD.

Studies have demonstrated decreased glucocorticoid levels in PTSD conditions, probably due to changes in the negative feedback activity of the associated gene receptors (Yehuda et al., 1991; Gold and Chrousos, 2002; Radley et al., 2011; Daskalakis et al., 2013). Our results are consistent with other studies regarding decreased Corticosterone in the PTSD rat model, which showed an increase at baseline, under stress, and during the return toward basal levels following Crocin treatment (Ettehadi et al., 2013). The ability of Crocin to inhibit Corticosterone changes in the current PTSD model indicate normalized HPA axis activity. It seems that the Corticosterone levels play an important role in the mechanism of PTSD.

To confirm the effect of Crocin in rats with PTSD, we evaluated changes in overt behaviors. Evaluating such behaviors in animal models may be used in therapeutic interventions. It has been reported that glucocorticoid in mesolimbic dopaminergic areas increases dopamine release and causes euphoric feelings and movements in rats (Howes et al., 2017). The increased freezing and decreased sniffing, rearing, and grooming behaviors in our study could be attributed to decreased Corticosterone levels in rats with PTSD.

We also observed a decreased incidence of freezing in crocin-treated rats, but no significant change was noted for other overt behaviors. Overt behaviors are those mediated by the dopaminergic system in the brain. Although physical movements (as opposed to freezing behavior) are directly controlled by the extra pyramidal dopamine system, they may be affected by glutamate, acetylcholine and gamma-aminobutryric acid neurotransmission systems (Shim et al., 2014). Since the anatomical locations of the behaviors in the brain are not similar, all behaviors may not be affected by the same intervention (medicine = Crocin, physical = stress) (Gottfried, 2006; Howes et al., 2017).

From cellular and molecular aspects, GluRs in the mesolimbic system play important roles in overt behaviors such as locomotion, rearing, and grooming (Howes et al., 2017). On the other hand, suppression of NMDARs receptors can decrease mesocorticolimbic system activity and ultimately inhibit brain dopamine and overt behaviors (Tzschentke, 2007). One study showed that suppression of GluR by MK-801 decreased movements in rats (Tzschentke, 2007). Similarly, suppression of dopamine receptors by sulpiride inhibits overt behaviors, indicating a close relationship between glutaminergic and dopamine pathways (Tzschentke, 2007). On the other hands, previous studies indicated that Crocin administration reduced the freezing time in the stress animals (Sahraei et al., 2012; Pitsikas, 2016). The reduced freezing time could be attributed to the reduced anxiety (Pitsikas, 2016). Interestingly, investigators insists that the Crocin can interact with NMDA receptors and act as it is antagonist (Hosseinzadeh et al., 2008; Berger et al., 2011). Hence, considering the inhibitory effect of Crocin on NMDARs, our results suggest a combinatorial mechanism by which Crocin decreases PTSD symptoms by influencing NMDAR activity and glucocorticoid levels.

In the present study, it has been demonstrated that crocin induces grooming (although it was not statistically significant), While in another study, it has been manifested that mCPP (a 5HT2c agonist) induced excessive self-grooming in the rat and crocin attenuated this effect of mCPP (Georgiadou et al., 2012); and in another study, crocin decreased grooming in mice (Hosseinzadeh and Noraei, 2009). These findings seem to be inconsistent with ours showing that crocin induces grooming. However, it should be noted that there is a remarkable difference between the procedures used in the mentioned studies. We have used the crocin alone as a treatment intervention in stressed animals, while the first study has used corcin against mCPP in animal model of obsessive–compulsive disorder (OCD) and, in the second study, crocin is used in intact animal.

Further advanced investigations into the relationships between different parts of the brain would be helpful to further elucidate this pathway.

We found no significant difference in the weights of the whole body or adrenal or thymus glands in PTSD rats with or without Crocin treatment compared with non-PTSD rats. Previous rodent studies reported that the chronic stress can cause weight loss and gain in males and females, respectively (Sato and Fahrenkamp, 2016). It seems that the present model, which had no significant effect on rat, is superior to those of the similar studies. Greater adrenal gland weight is one of the most important effects of chronic stress and results from hyperplasia in the fascicular zone of the adrenal cortex (Bali and Jaggi, 2015), which stimulates the HPA to have a more robust response to stress. Increased adrenal gland weight was not observed in the present study, possibly due to decreased sensitivity of the HPA axis. Others have reported smaller thymus size following chronic stress due to the inhibitory effect of glucocorticoids on T-cell differentiation (Pertsov et al., 2015). Again, these results were not observed in our acute stress model.

The question which might be asked is that if the animals' behavior and/or other experimental procedures were evaluated in different days (other than day 21), different results would be obtained. It is an important issue which needs to be further investigated in the future experiments. Indeed, considering various times of interventions and their results would be of importance, and it must be considered for future experiments. However, in the present study, the protocol used by was noticed (Mikics et al., 2008; Zoladz et al., 2012).

## Conclusion

The psychoactive agent Crocin possesses glutamate receptor agonist properties that can regulate HPA axis activity and has the potential to reduce PTSD symptoms. The model employed in the current study is applicable for evaluating HPA axis disorders.

## Author contributions

On behalf of the corresponding author I would like to undertake the responsibility that all authors have contributed in this study in terms of experimental work, statistical analysis, scientific writing and revision. SA, MT, HS, and GP contributed to the design of the study, experimental work, drafting of the paper, major revisions, and final approval for publication.

### Conflict of interest statement

The authors declare that the research was conducted in the absence of any commercial or financial relationships that could be construed as a potential conflict of interest.
